# Fish oil, lard and soybean oil differentially shape gut microbiota of middle-aged rats

**DOI:** 10.1038/s41598-017-00969-0

**Published:** 2017-04-11

**Authors:** He Li, Yingying Zhu, Fan Zhao, Shangxin Song, Yingqiu Li, Xinglian Xu, Guanghong Zhou, Chunbao Li

**Affiliations:** 1 Key Laboratory of Meat Processing and Quality Control, MOE, Key Laboratory of Meat Processing, MOA, Jiang Synergetic Innovation Center of Meat Processing and Quality Control, Nanjing Agricultural University, Nanjing, 210095 P.R. China; 2 School of Food Science, Nanjing Xiaozhuang University, Nanjing, 211171 P.R. China

## Abstract

High-fat diets have been associated with overweight/obesity and increased mortality in middle-aged populations. However, it is still unclear how gut microbiota in middle-aged populations responds to dietary fats at a normal dose. In this study, we explored gut microbiota structure in middle-aged rats (aged 12 months) after feeding 4% (w/w) soybean oil, lard or fish oil for 3 months, respectively. The results showed that the gut microbiota structure in the fish oil group was substantially different from those of the soybean oil and lard groups in both *in vitro* and *in vivo* studies. The relative abundances of phylum *Proteobacteria* and genus *Desulfovibrio* in the caecal and colonic contents were the highest in the fish oil group (p < 0.05). The mRNA levels of biomarkers for inflammation in the colon, including IL-1β, IL-6, IL-17, IL-18 and TNF-α, were also the highest in the fish oil group (p < 0.05). Meanwhile, the fish oil group had the highest microbial DNA abundance of a predicted lipid metabolism. Our results gave a new insight into the potentially negative impact of fish oil diet on health of middle-aged populations by changing gut microbiota and inducing inflammation as compared to soybean oil and lard diets.

## Introduction

In recent years, high-fat diets have been associated with overweight/obesity and increased mortality in middle-aged populations and the Mediterranean diet containing olive oil may reduce the risk to mortality at middle ages^1, 2^. Middle ages are considered an age range from 45 to 65 years old for human and from 10 to 14 months old for rats^3, 4^, during the period of which metabolic disorders may happen and further affect health at the elderly stages^5, 6^. And thus it is extremely important to keep a healthy status at middle ages so as to realize an extended and healthy lifespan. It is widely known that diet fats have a great influence on the occurrence of some metabolic syndromes^7, 8^. Fat is an important nutrient that is either derived from diet or transformed from carbohydrates and proteins. Dietary fats vary greatly in fatty acid composition. For example, pork lard and beef fat contain high amounts of saturated fatty acids (SFA), while fish oil is composed of higher levels of n-3 polyunsaturated fatty acids (PUFA). Previous studies have shown that high intake of saturated fatty acids is more likely to result in increased intestinal permeability, insulin resistance, impaired gut barrier function and increased adipocyte hyperplasia in white adipose tissue^9–11^. In contrast, diets rich in polyunsaturated fatty acids would enhance epithelial resistance and membrane integrity^12^. The fatty acid composition in diets has been shown to have a great impact on fat bioavailability in the small intestine under the emulsifying functions of bile acids^13, 14^. Bile acids act as signaling molecules and intermediates between the host and the gut microbiota^15^.

Dietary fats may affect host health by altering gut microbial communities^16^. There are trillions of microbes in the human intestine, amounting to greater than 100-fold prokaryote genes of the host^17, 18^. The gut microbiota structure is associated with gut health^16^. High abundances of commensal bacteria in gut would enhance metabolic capabilities of the gut and protect the gut against pathogens^19–21^. Western diets characterized by high fats were shown to reduce the diversity of gut microbiota, which could be associated with obesity and inflammatory bowel disease^22–24^. Nevertheless, it is fatty acid composition rather than the intake amount that is relevant to metabolic disorders^25^. In 11-to-14-week-old mouse studies, high-lard diets were observed to reduce phylogenetic diversity and abundance of lactobacillus as compared to normal diets^25^, but increase the abundance of sulfate/sulfite-reducing bacteria as compared to high-fish-oil diets^26, 27^. However, to our knowledge, it remains unclear how gut microbiota in middle-aged populations responds to different type of fat diets.

The present study was to explore the responses of gut microbiota of middle-aged rats to intake of lard, fish oil and soybean oil. An *in vitro* study by incubating rat feces with the above three fats was also performed and the possible mechanism was discussed.

## Results

### *In vivo* study

#### Overall profiles of gut microbiota in response to different fat diets

On the operation taxonomy unit (OTU) level, 29,170 OTUs were detected from all 33 rats. UniFrac principal co-ordinate analysis showed great differences between the fish oil group and the soybean oil group for both caecal and colonic samples. The lard group and the soybean oil group could not be well separated **(**Supplementary Fig. 1). This indicated that the fish oil diet had a different impact on gut microbiota of middle-aged rats from the lard and soybean oil diets.

**Figure 1:**
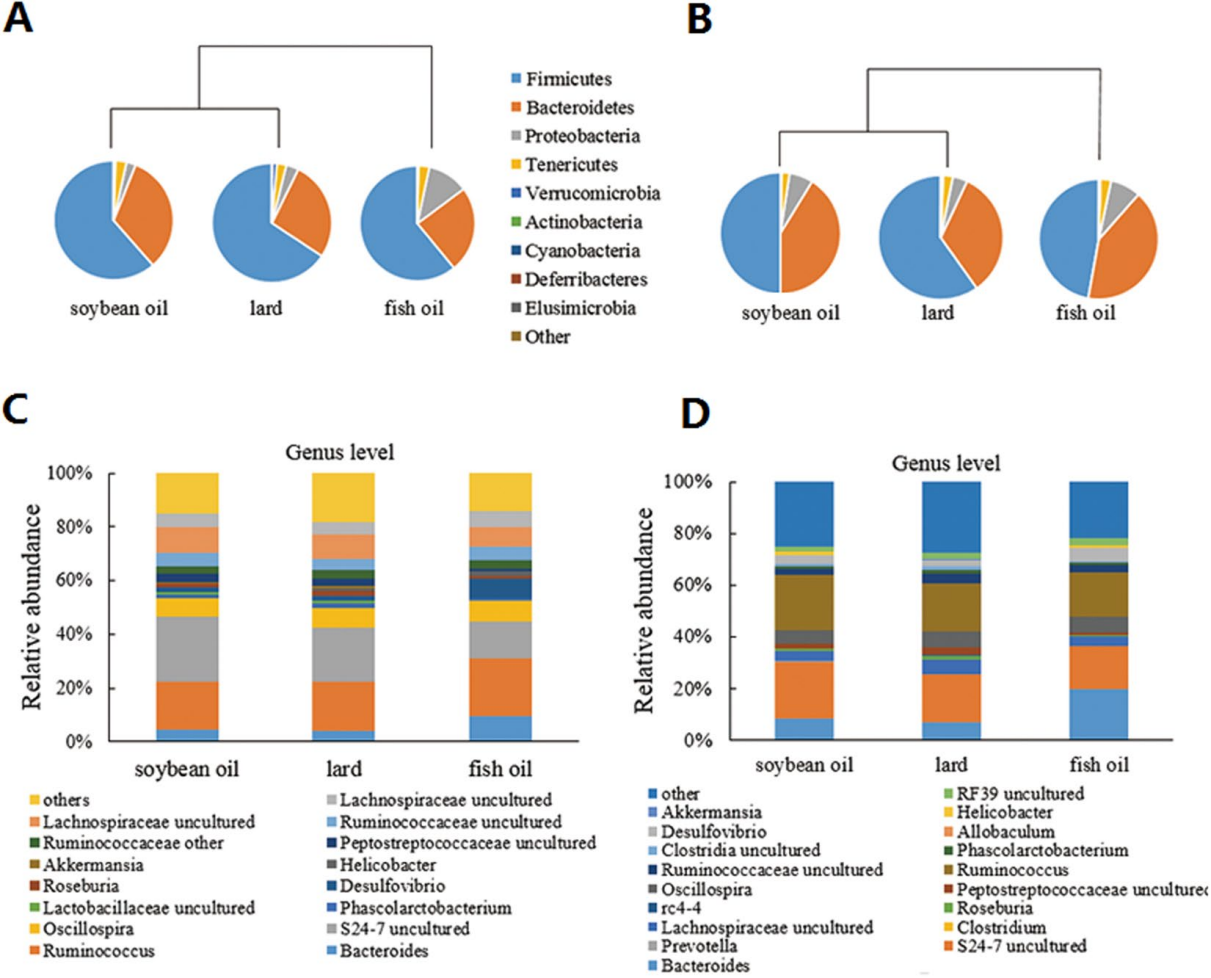
Gut microbiota in response to dietary fats. (**A**) Relative abundance of caecel microbiota on the phylum level. (**B**) Relative abundance of colonic microbiota on the phylum level. (**C**) Relative abundance of caecal microbiota on the genus level. (**D**) Relative abundance of colonic microbiota on the genus level.

On the phylum level, the caecal and colonic samples of the fish oil group could be separated from those of the soybean oil group and the lard group (Fig. 1A and B). *Firmicutes* and *Bacteroidetes* constituted the vast majority of caecal microbiota for all the three diet groups ranging from 82% to 93%. The ratio of *Firmicutes* to *Bacteroidetes* was the highest for the lard group (3.27 ± 1.47, 3.01 ± 1.96, and 2.20 ± 1.02 for the lard, fish oil and soybean oil groups, respectively). The fish oil group had a significantly higher abundance of *Proteobacteria* than the other two groups (p < 0.05). *Verrucomicrobia* and *Tenericutes* were more abundant in the lard group (p < 0.05) than those in the fish oil group and the soybean oil group. All the other phyla did not differ in abundance among the three diet groups (p > 0.05). In addition, colonic content samples had higher percentages of *Bacteroidetes* but lower *Firmicutes* than caecal content samples (Fig. 1A versus B).

On the genus level, both caecal and colonic content samples of the fish oil group had higher abundance of *Desulfovibrio* but lower abundances of *Roseburia* and *Phascolarctobacterium* than the other two groups (p < 0.05, Fig. 1C and D). *Akkermansia* was more abundant in the lard group as compared to the fish oil and soybean oil groups (p < 0.05). *Helicobacter* in colonic content samples was observed more abundant in the soybean oil group (p < 0.05, Fig. 1C), but it was more abundant in caecal content samples of the fish oil group (p < 0.05, Fig. 1D).

#### High-dimensional biomarkers for differentiating gut bacteria on the OTU level

**Figure 2:**
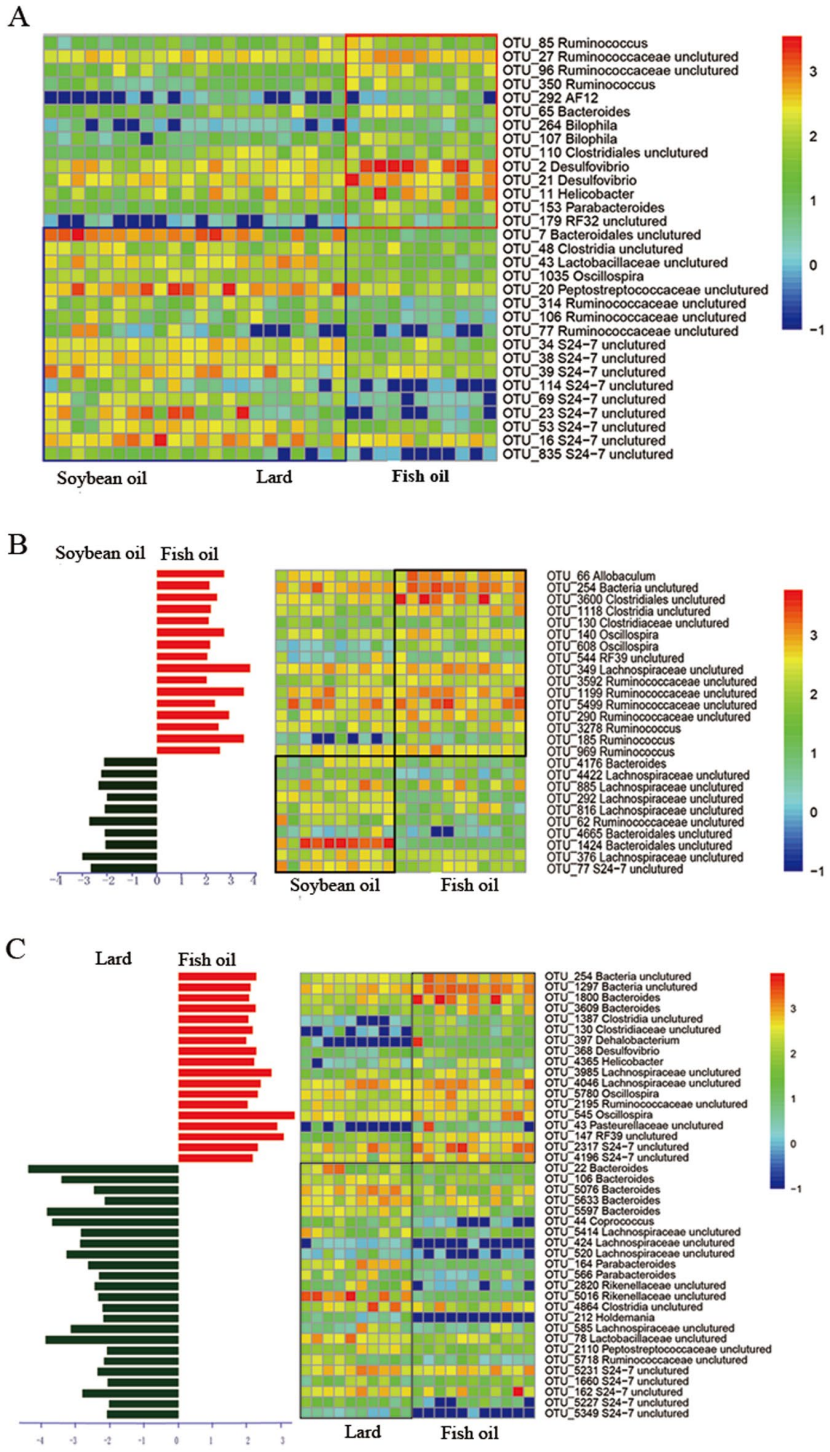
Comparisons of gut bacteria on OTU level using LefSe (**A**) caecal bacteria; (**B**) colonic bacteria between soybean oil and fish oil; (**C**) colonic bacteria between lard and fish oil. In **B** and **C**, the left histograms show the LDA scores computed for features on the OTU level. The middle heatmaps show the relative abundance of OTU (log 10 transformed). Each column represents one biological sample and each row represents the OTU corresponding to left one.

LefSe analysis was performed to identify phylotypes that were sensitive to fat diets and had the relative abundance of OTUs greater than 0.05% at least in one diet group. On the OTU level, 31 phylotypes were identified in caecal content samples as high dimensional biomarkers (Fig. 2A). Fourteen of these phylotypes were more abundant in the fish oil group (p < 0.01) than those in the lard group and the soybean oil group, including genera *Bilophila* (OTU264 and OTU107), *Desulfovibrio* (OTU2 and OTU21) and *Helicobacter* (OTU11). The other 17 OTUs were less abundant in the fish oil group (p < 0.05), including genera *Peptostreptococcaceae uncultured* (OTU20), *Oscillospira* (OTU1035) and *S24-7 uncultured* (OTU34, OTU38, OTU39, OTU114, OTU69, OTU23, OTU53, OTU16 and OTU835). We did not find microbial biomarkers in the caecal content samples that distinguish the lard group from the soybean oil group.

In colonic content samples, 26 phylotypes were observed different between the soybean oil group and the fish oil group (Fig. 2B). Ten of these phylotypes were more abundant in the soybean oil group, including *Bacteroides* (OTU4176) and *Bacteroidales uncultured* (OTU1424). However, the remaining 16 phylotypes were more abundant in the fish oil group, including *Oscillospira* (OTU140 and OTU608), *Allobaculum* (OTU66) and *Ruminococcus* (OTU3278, OTU185 and OTU969). Furthermore, 42 biomarkers were found between the lard group and the fish oil group (Fig. 2C). *Bacteroides* (OTU1800 and OTU3609), *Bacteria uncultured* (OTU254 and OTU1297), *Desulfovibrio* (OTU368) and *Oscillospira* (OTU5780) were more abundant in the fish oil group, but the lard group had higher abundance of *Parabacteroides* (OTU164 and OTU566). No microbial biomarkers were found in the colonic content samples between the soybean oil group and the lard group.

#### Diet-induced changes of physiological responses

**Figure 3:**
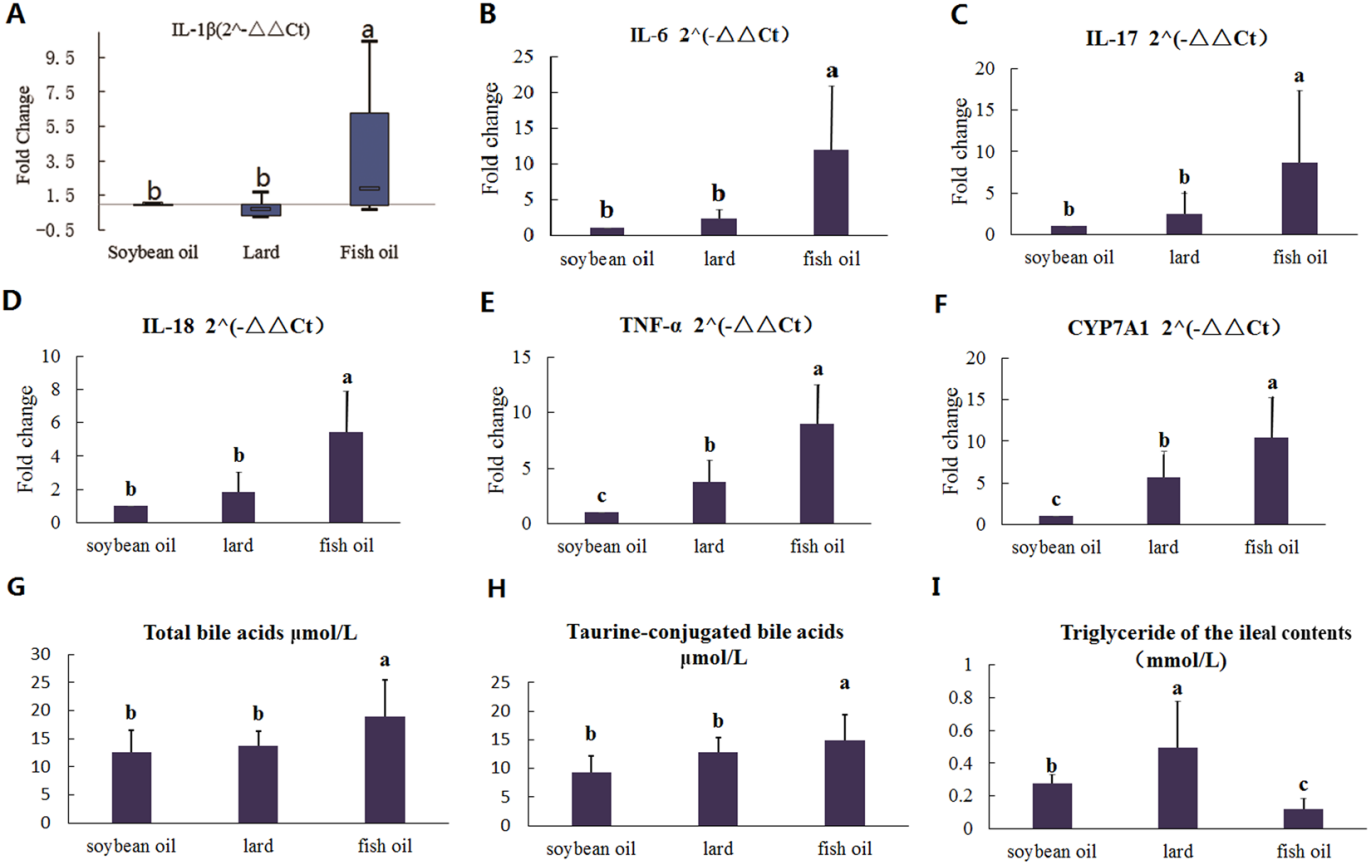
Diet-induced changes of physiological parameters of the rats. (**A–E**) The expression of inflammatory cytokines in the colon tissue, including IL-1β (**A**), IL-6 (**B**), IL-17 (**C**), IL-18 (**D**) and TNF-α (**E**). (**F**) The expression of gene CYP7A1 in the liver. (**G**) The amounts of total bile acids in the caecal contents. (**H**) The amounts of taurine-conjugated bile acids in the caecal contents. (**I**) The amounts of triglyceride in the ileal contents The data were analyzed by one-way analysis of variance and means were compared by the procedure of Duncan’s multiple-range comparison. a,b,c, means with different letters differed significantly (p < 0.05). Error bar means standard deviation.

Gut microbiota has been recognized to be associated with host physiology and colonic health. To evaluate the impact of fat diets on colonic health, in particular to inflammation, qPCR was performed to quantify the mRNA levels of inflammation biomarkers including IL-1β, IL-6, IL-17, IL-18 and TNF-α in the colonic tissue (Fig. 3A to E). The results indicated that IL-1β, IL-6, IL-17 and IL-18 were highly expressed in the fish oil group as compared to those in the soybean oil group (p < 0.05, Fig. 3A–D), but no differences were observed between the lard group and the soybean oil group (p > 0.05). The TNF-α levels were higher in the fish oil group and the lard group than that of the soybean oil group (p < 0.05, Fig. 3E).

The above results indicate that the fish oil diet may potentially increase the risk to inflammation. This could result from the differences in fatty acid composition and cholesterol level in diets (Supplementary Table 1). Fish oil had the highest contents of cholesterol and total n-3 PUFAs. And the total SFA content in fish oil was higher than that in soybean oil, but lower than that in lard. The metabolic end products of cholesterol in the liver are bile acids that are key carrier for fat absorption in the intestine. Cholesterol 7α-hydroxylase (CYP7A1) plays a critical role in the formation of bile acids in the liver. Triglyceride content in the ileal contents was the lowest in the fish oil group but the highest in the lard group (p < 0.05, Fig. 3I). Meanwhile the amounts of total bile acids and taurine-conjugated bile acids in caecal contents were the highest in the fish oil group (p < 0.05, Fig. 3G and H). In addition, CYP7A1 mRNA level in the liver was also the highest in the fish oil group (p < 0.05, Fig. 3F). These results indicated that fish oil could be more efficiently absorbed in the intestine by upregulating CYP7A1 expression and bile acid production.

In both caecal and colonic content samples, total SCFAs, acetic acid and propionic acid concentrations were lower in the lard group than those in the soybean oil group and the fish oil group (p < 0.05, Table 1). No significant difference was observed in these variables between the soybean oil group and the fish oil group (p > 0.05). On the other hand, the lard group had higher butyrate acid in caecal contents than the other two groups (p < 0.05). The differences in SCFAs among diet groups could be associated with gut microbiota.

**Table 1:** SCFAs levels in the cecum and colon in response to different dietary fats (mmol/L).

	acetic acid	propionic acid	isobutyric acid	butyrate acid	isovaleric acid	valeric acid	Total SCFAs
Caecal contents							
Soybean oil	47.07 ± 8.73^*a*^	5.77 ± 0.91^*a*^	1.83 ± 0.72^*a*^	7.75 ± 2.37^*ab*^	1.82 ± 0.41^*a*^	1.90 ± 0.33^*a*^	66.15 ± 11.35^*a*^
Lard	32.88 ± 12.35^*b*^	3.71 ± 0.73^*b*^	2.92 ± 4.71^*a*^	8.97 ± 1.72^*a*^	1.44 ± 0.21^*b*^	1.83 ± 0.25^*a*^	51.76 ± 15.47^*b*^
Fish oil	47.40 ± 12.58^*a*^	5.74 ± 1.11^*a*^	1.56 ± 0.77^*a*^	6.34 ± 1.15^*b*^	1.60 ± 0.46^*ab*^	1.82 ± 0.31^*a*^	64.56 ± 12.85^*a*^
Colonic contents							
Soybean oil	42.88 ± 12.84^*a*^	4.04 ± 1.38^*a*^	1.11 ± 0.68^*a*^	5.24 ± 2.55^*a*^	1.15 ± 0.34^*a*^	1.11 ± 0.32^*a*^	55.54 ± 15.74^*a*^
Lard	16.75 ± 7.43^*b*^	1.81 ± 0.74^*b*^	1.02 ± 0.49^*a*^	3.57 ± 1.59^*a*^	1.02 ± 0.25^*a*^	0.91 ± 0.29^*a*^	25.08 ± 10.06^*b*^
Fish oil	41.24 ± 23.04^*a*^	4.26 ± 2.54^*a*^	1.47 ± 0.77^*a*^	4.57 ± 1.24^*a*^	1.06 ± 0.33^*a*^	1.12 ± 0.36^*a*^	53.73 ± 23.64^*a*^

^*a*,*b*^Means with different superscripts differed significantly (P < 0.05).

The numerical value is composed of the mean and the standard deviation.

### *In vitro* study

To partially verify the associations between the type of dietary fats and the composition of gut bacteria, an *in vitro* study was performed, in which the caecal microbiota was incubated with fat digesta.

**Figure 4:**
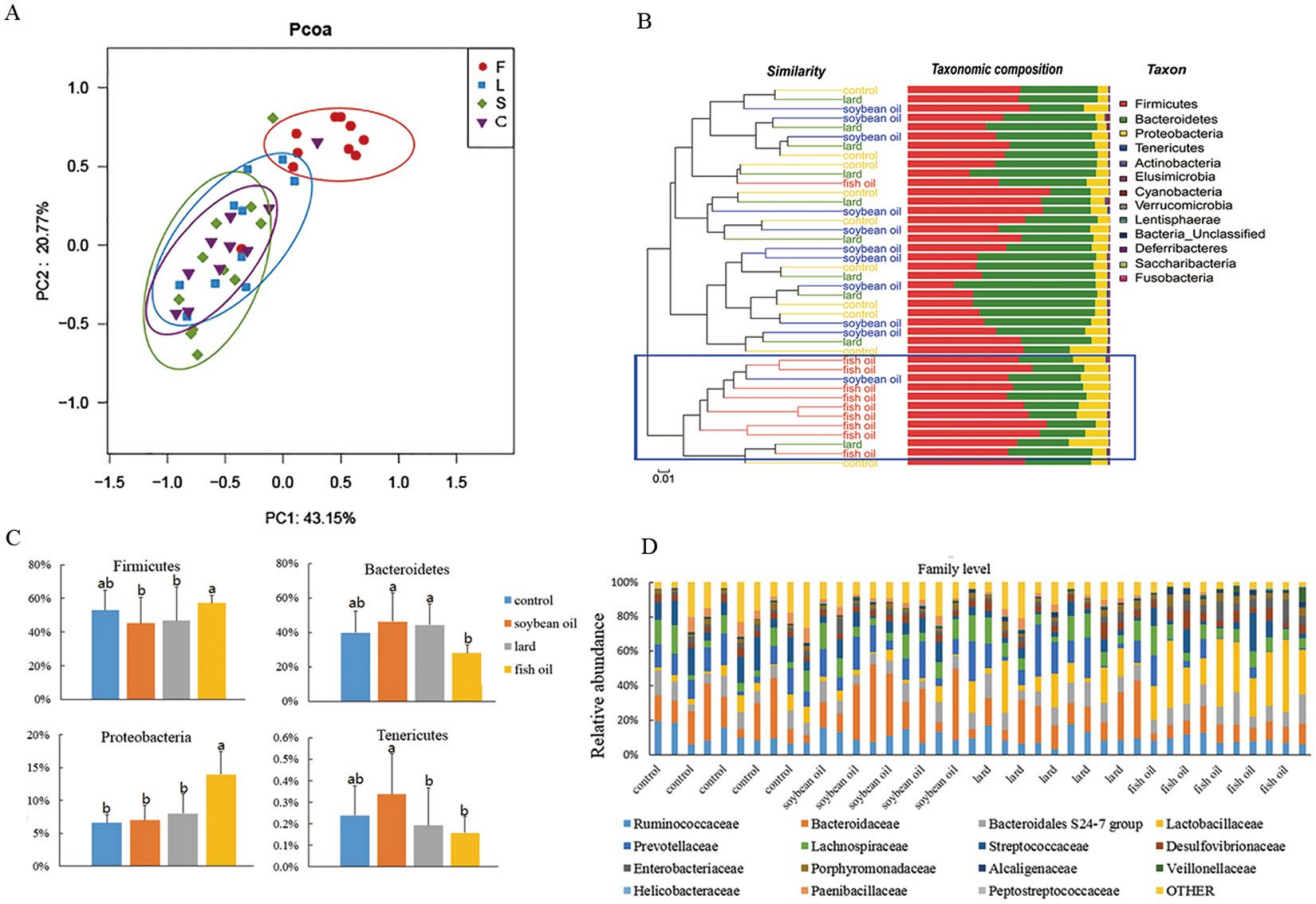
Composition of microbiota in fermentation liquids. (**A**) Principal coordinate analysis of fermentation liquids on the OTU level. F, L, S and C represent fish oil, lard, soybean oil and control groups, respectively. (**B**) Relative abundance of microbial community at the phylum level in fermentation liquids in response to three dietary fats. (**C**) Changes of relative abundance of phyla with significant difference in fermentation liquids. Data were shown as means and standard deviation (bars). The data were analyzed by one-way analysis of variance and means were compared by the procedure of Duncan’s multiple-range comparison. a, b, means with different letters differed significantly (p < 0.05). (**D**) Relative abundance of microbial community in fermentation liquids on the family level.

Under the 16S rDNA sequencing platform, the overall microbial composition in fermentation liquids was compared. On the OTU level, the principal coordinate analysis showed a distinct separation of the fish oil group from the lard group and the soybean oil groups (Fig. 4A). However, the lard group and the soybean oil group did not show a great difference. This is in accordance with the results of the *in vivo* study.

Clustering analysis also showed that the fish oil group differed from the lard group and the soybean oil group (Fig. 4B and C). On the phylum level, *Firmicutes* and *Bacteroidetes* were the two predominant phyla in the fermentation liquids. The fish oil group had the highest abundances of *Firmicutes* and *Proteobacteria*, but the lowest *Bacteroidete*s and *Tenericutes* (p < 0.05, Fig. 4C).

On the family level, the fish oil group had higher abundances of *Lactobacillaceae* and *Desulfovibrionaceae* but lower *Prevotellaceae* (p < 0.05, Fig. 4D). The lard group had higher abundance of *Enterobacteriaceae* (p < 0.05, Fig. 4D).

**Figure 5:**
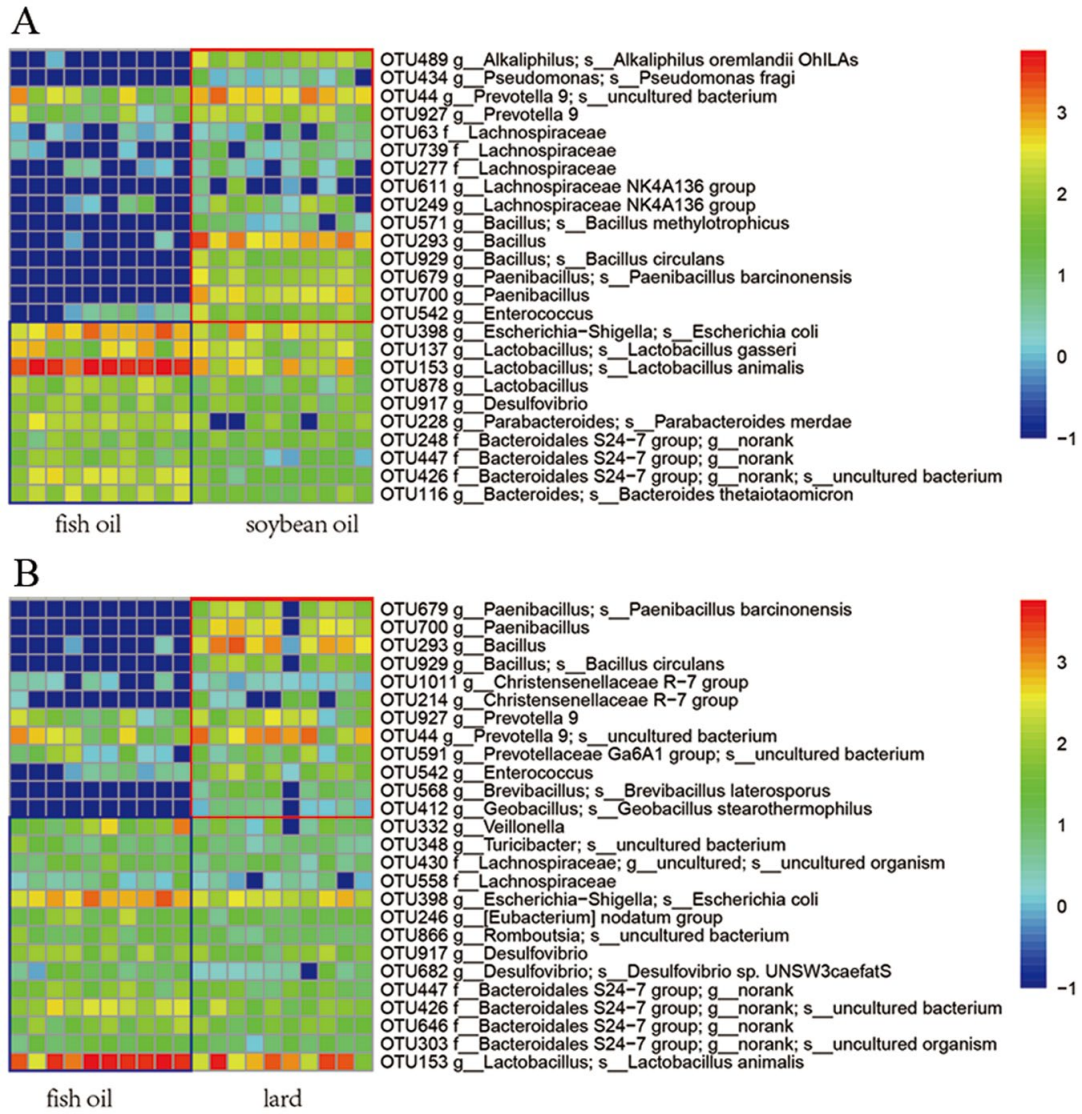
Comparisons of bacteria in fermentation liquids using LefSe. (**A**) Between fish oil and soybean oil groups. (**B**) Between fish oil and lard groups. The left histograms show the LDA scores computed for features on the OTU level. The heatmaps shows the relative abundance of OTU (log 10 transformed). Each column represents one biological sample and each row represents the OTU corresponding to the right one.

On the OTU level, 25 phylotypes were observed to discriminate the soybean oil group from the fish oil group (Fig. 5A). Fifteen of these OTUs were more abundant in the soybean oil group (p < 0.05), including genera *Prevotella* (OTU927 and OTU44), *Lachnospiraceae uncultured* (OTU63, OTU277 and OTU739), and *Lachnospiraceae NK4A136* (OTU249 and OTU611). The remaining 10 OTUs were more abundant in the fish oil group (p < 0.05), including *Lactobacillus* (OTU137, OTU153 and OTU878) and *Desulfovibrio* (OTU917).

Twenty-six OTUs were observed different between the lard group and the fish oil group (Fig. 5B). Of them, 12 OTUs were more abundant in the lard group (p < 0.05), including *Prevotella 9* (OTU927 and OTU44), *Prevotellaceae Ga6A1 group* (OTU591) and *Enterococcus* (OTU542). The fish oil group had higher abundances (p < 0.05) of *Lactobacillus* (OTU153), *Desulfovibrio* (OTU917 and OTU682) and *S24-7 uncultured* (OTU303, OTU646, OTU426 and OTU447).

**Figure 6:**
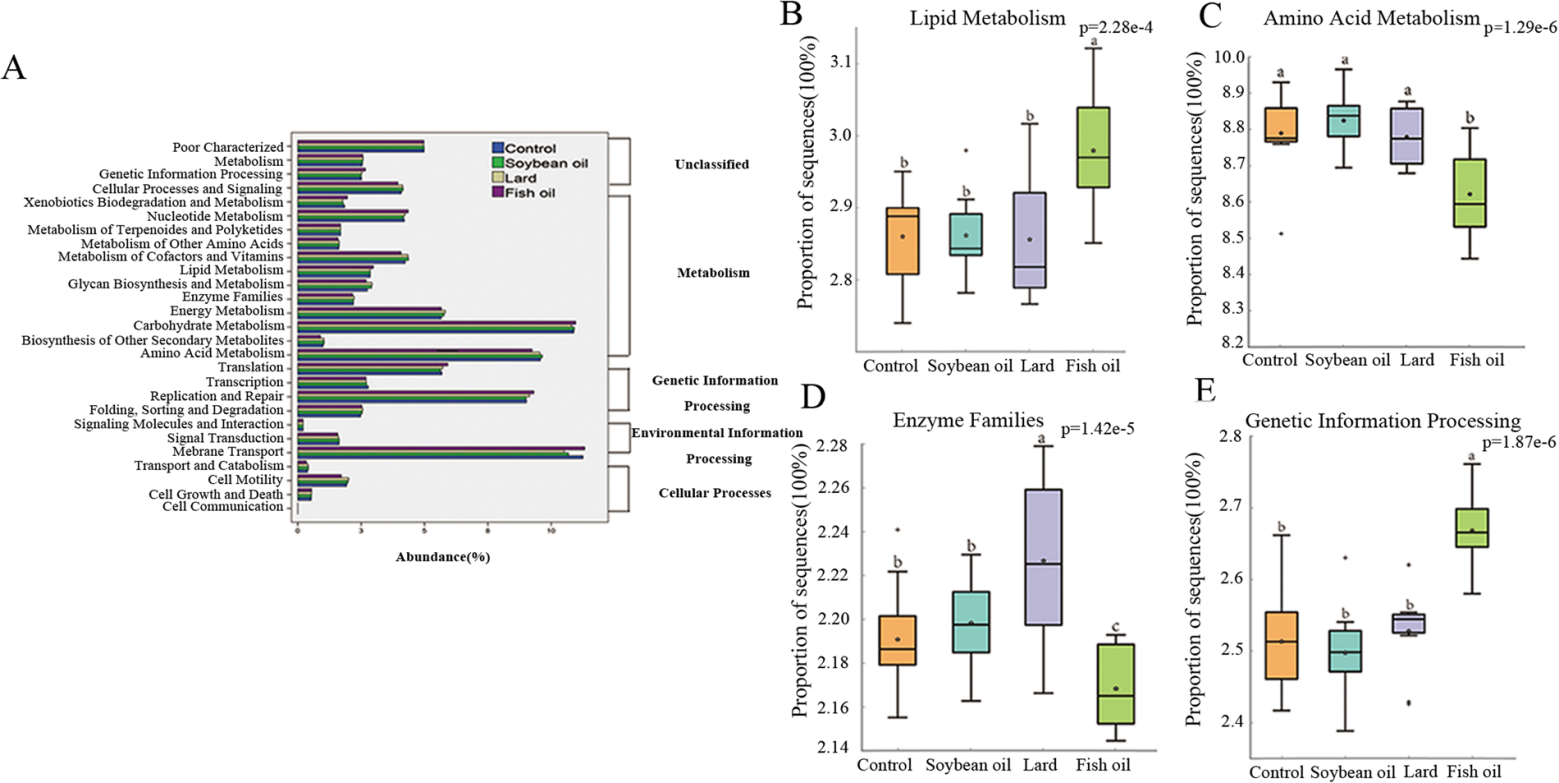
PICRUSt recapitulates biological findings. (**A**) Abundances of KEGG pathways in level-2 functional prediction by PICRUSt. (**B**) Lipid metabolism. (**C**) Amino acid metabolism. (**D**) Enzyme families. (**E**) Genetic information processing. The data were analyzed by one-way analysis of variance and means were compared by the procedure of Duncan’s multiple-range comparison. a,b,c means with different letters differed significantly (p < 0.05).

The predictive functions of the microbiota genes were shown in Fig. 6. “Metabolism” was the most dominant category, of which amino acid metabolism, carbohydrate metabolism and energy metabolism were the top three pathways (Fig. 6A). The major different functions include the metabolism of amino acids, carbohydrates and lipids, enzyme families and genetic information processing on the second KEGG level (Fig. 6B to E). Incubation of caecal bacteria with fish oil induced higher abundances of predicted lipid metabolism and genetic information processing (p < 0.05), but lower abundances of predicted amino acid metabolism and enzyme families (p < 0.05). On the corresponding third KEGG level, the abundances of predicted metabolism of bile acids and fatty acids, and DNA repair and recombination proteins were higher in the fish oil group than those of the lard group and the soybean oil group, while the abundances of predicted amino acid related enzymes were the lowest in the fish oil group (Supplementary Fig. 3, p < 0.05). The predictive functions of the caecal bacteria in the *in vivo* study were similar to those of the *in vitro* study (Supplementary Fig. 4).

By comparing gut microbiota structure between *in vivo* and *in vitro* studies, we found that 129 OTUs (mainly belonging to genera *Bacteroides* and *Ruminococcus*) were common for *in vivo* samples and *in vitro* samples (Supplementary Fig. 2). However, 145 OTUs were only detected in the caecal content samples (including genera *Phascolarctobacterium* and *AF12*), while 103 OTUs were only detected in fermentation liquids. This indicates that 145 OTUs found in *in-vivo* samples could not be cultured in the *in vitro* conditions. On the other hand, 103 OTUs found in fermentation liquids may be inhibited in the animal gut but they could grow well if the conditions were altered.

The short chain fatty acid composition in the *in-vitro* conditions showed a great difference from the *in vivo* conditions (Table 2). The concentrations of acetic, propionic and butyrate acids in fermentation liquids were substantially higher than those of caecal contents, however, the concentrations of isobutyric and isovaleric acids in fermentation liquids were slightly lower than those in caecal contents. There was significant difference only in acetic acid concentration between the fish oil group and the lard group (p < 0.05, Table 2).

**Table 2:** SCFAs levels in the fermentation liquid in response to different dietary fats (mmol/L).

	acetic acid	propionic acid	isobutyric acid	butyrate acid	isovaleric acid	valeric acid	Total SCFAs
Control	205.10 ± 27.97^*a*^	31.25 ± 5.19^*a*^	0.59 ± 0.32^*ab*^	34.77 ± 10.67^*a*^	0.85 ± 0.94^*ab*^	2.07 ± 1.56^*a*^	274.62 ± 27.75 ^*a*^
Soybean oil	179.63 ± 35.41^*ab*^	39.56 ± 20.33^*a*^	0.77 ± 0.51^*a*^	30.89 ± 10.15^*a*^	1.13 ± 1.25^*a*^	1.51 ± 0.99^*a*^	274.85 ± 39.93^*a*^
Lard	162.30 ± 37.72^*b*^	33.00 ± 14.33^*a*^	0.49 ± 0.16^*b*^	30.95 ± 11.99^*a*^	0.59 ± 0.16^*ab*^	1.29 ± 0.95^*a*^	228.63 ± 49.62^*b*^
Fish oil	203.37 ± 32.55^*a*^	34.90 ± 13.78^*a*^	0.48 ± 0.12^*b*^	33.72 ± 11.45^*a*^	0.51 ± 0.15^*b*^	1.87 ± 1.49^*a*^	253.49 ± 52.47^*ab*^

^*a*,*b*^Means with different superscripts differed significantly (P < 0.05).

The numerical values were composed of the means and the standard deviations.

## Discussion

Age affects both the composition and function of the gut microbiome in mice^28^. Much attention has been paid to the diet effect on gut microbiota of infant, young and elderly populations^29–31^. Previous studies have shown that *Bifidobacterium longum*, *B. breve*, and *B. bifidum* are generally dominant in infants, whereas *B. catenulatum* and *B. adolescentis* are more prevalent in adults^32^. In human, significant differences have been observed in gut microbiota structure between the adults and the elderly^33^. The diversity of gut bacteria decreases as age increases^33^. In addition, the ratio of *Firmicutes* to *Bacteroidetes* was lower in elderly people than in infants^33^. In the *Firmicutes*, *Clostridium cluster XIVa* was reported to decrease in elderly people^34^. However, as the most important part of a lifespan, gut microbiota of middle-aged populations has been less studied in terms of diet effect.

Fat accounts for approximately 30–35% of the total energy intake in western diets^35^. Fatty acid composition has been shown to affect the digestion and absorption of dietary triglycerides^13^. In fact, the amount of triglycerides reaching the caecum is influenced by the type of dietary fats. In the *in vivo* study, the amount of triglycerides in the ileal contents was the highest in the lard group but the lowest in the fish oil group, as it is in the caecum (Supplementary Table 2), indicating that fish oil can be more efficiently absorbed than lard in the small intestine. This may partially explain the differences in gut bacteria structure in caecal contents among the three diet groups and further shape the gut bacteria in the colonic contents. The ratios of *Firmicutes* to *Bacteroidetes* were ranked as “the lard group > the fish oil group > the soybean oil group” in both caecal and colonic contents. Although the ratio of *Firmicutes* to *Bacteroidetes* has been associated with body weight and metabolic disorders^22, 34–36^, none of the rats showed any phenomenon of metabolic disorders in the present study. Most importantly, the lard group had a greater weight gain than the fish oil group and the soybean oil group even if the daily feed intake did not differ between any two groups (Supplement Table 2). This could be because relatively high intake of SFA may induce higher abundance of the phylum *Firmicutes* ^23^.

On the other hand, intake or incubation of fish oil led to a substantially high population of the phylum *Proteobacteria* in both *in vivo* and *in vitro* studies. This finding is consistent with earlier studies showing higher abundance of *Proteobacteria* in mice fed high fish oil diet^25^. This result could be attributed to the differences in fatty acid composition in fat diets. Long-chain n-3 PUFA is widely advised for public health^37, 38^. However, PUFA are highly prone to oxidation, and their oxidation end-products, 4-hydroxy-2-alkenals (4-HHE), are considered to cause metabolic oxidative stress and inflammation^39–41^. The absorption of 4-HHE in the small intestine was associated with the formation of 4-HHE-protein adducts and increased expression of glutathione peroxidase 2 and glucose-regulated protein 78^42–44^. In the previous study, intestinal inflammation was positively correlated with the abundance of *Proteobacteria* ^45^. And thus it is inferred that the oxidation of PUFAs in fish oil could induce the higher abundance of *Proteobacteria* in the fish oil group. In addition, the fish oil group had higher abundances of *Desulfovibrionaceae* and *Bilophila*. This could result from a higher level of cholesterol in fish oil. It is known that cholesterol is transformed into bile acids in the liver, and bile acids are excreted to the small intestine and play an important role in fat emulsification and absorption^14^. Formation of bile acids is complex and includes several reaction steps catalyzed by some enzymes^46^. CYP7A1 is one of the rate-limiting enzymes that affect the production of bile acids^39^. In the present study, the mRNA level of CYP7A1 in the liver was the highest in the fish oil group and the lowest in the soybean oil group. This is because cholesterol does not exist in soybean oil. Meanwhile, low levels of bile salts may increase the activity of pancreatic triacylglycerol lipase, but they would inhibit the enzymatic activity at higher concentrations^40^. The majority of bile acids are conjugated with taurine and glycine^41^. In the present study, total bile acids and taurine-conjugated bile acids in the caecal contents were higher in the fish oil group. Higher amounts of taurine-conjugated bile salts could stimulate the growth of sulfite/sulfate reducing bacteria including *Desulfovibrionaceae* and *Bilophila* ^47^. This could be confirmed by the higher abundance of predicted metabolism of bile acids on the third KEGG level in the fish oil group. High hydrogen sulfide may break the metabolic balance and cause endotoxemia, which would increase pathogen adhesion to intestinal wall, impair intestinal barrier function and finally increase the risk to inflammation^47^. High hydrogen sulfide can also reduce butyrate oxidation that is critical to control colonocyte turnover^48^. PICRUSt function prediction further indicated that the fish oil group had a higher capacity of lipid metabolism. This is because PUFAs could be more difficult to combine with bile acids and less effectively transported and utilized than SFAs and mono unsaturated fatty acids^49^. Quantitative RT-PCR revealed that the fish oil group exhibited higher levels of IL-1β, IL-6, IL-17, IL-18 and TNF-α in colon tissue. These interleukins play an important role in immune cell differentiation and activation, however high levels of these interleukins may also induce inflammation^50^. Taken together, intake of fish oil may increase the taurine-conjugated bile acids which promote the growth of *Proteobacteria*, and the bacterium produce hydrogen sulfide to induce gut inflammation. And thus three-month intake of fish oil may induce inflammation in colon partially because of the alteration of gut bacteria.

SCFAs serve as energy source and physiological and immune regulators of the host^51^. The present study showed a significant impact of dietary fats on SCFAs in both *in vivo* and *in vitro* studies. In both conditions, the lard group had lower acetic acid than the fish oil group. This result coincided with the lower abundance of predicted carbohydrate metabolism in the lard group. Therefore, intake of lard diet may reduce the abundances of gut bacteria that utilize polysaccharide and oligosaccharides. On the other hand, the level of butyrate acid, an important energy source for gut bacteria and epithelial cells, was the highest in the lard group. The reason could be that lard contains decanoate acid (C10:0) that has capacity of increasing the thickness of intestinal mucosa and improving intestinal ecosystem and permeability^52^. Soybean oil and fish oil do not have such medium-chain fatty acids.

*Akkermansia* is considered as a beneficial gut bacterium that can stimulate the growth of the large intestinal mucus layer and protect the host against metabolic endotoxemia, obesity and systemic inflammation^53^. In a high-fat diet study, Caeser *et al*. (2015) found that intake of high-lard diet (40% kcal fat) decreased the abundance of genus *Akkermansia* as compared to high-fish-oil diet^25^. However, a higher abundance of *Akkermansia* was observed in the lard group (10% kcal fat in diet) as compared to the soybean oil group and the fish oil group in the present study. The difference between the two studies could be attributed to the fat amounts in diets (40% kcal in fat for Caeser *et al*. versus 10% kcal in fat for the present study). However, its underlying mechanism needs further study.

By comparing microbial composition between *in vivo* and *in vitro* studies, nearly half number of identified gut bacteria could not be cultured in the *in vitro* study. On the other hand, many very low abundant gut bacteria can be well cultured in the *in vitro* condition. Gut bacteria that can be grown *in-vitro* are only a fraction of the total diversity that exists in *in-vivo* conditions^54^. And uncultured bacteria that do not grow in *in-vitro* conditions are playing critical roles in synthesis of novel natural products, and in turn affecting the surrounding organisms and environment^54^.

In summary, we explored the impact of dietary fats on gut microbiota composition and several physiological aspects of middle-aged rats and found that the type of dietary fats had a significant impact on gut microbiota and physiological responses at a normal dose (4% fat in diet, amounting to 10% kcal in fat). Intake of fish oil increased *Desulfovibrionaceae* abundance and microbial capacity of lipid metabolism in caecal contents, which was accompanied with higher levels of IL-1β, IL-6 and TNF-α in colonic tissue and CYP7A1 in the liver. However, intake of lard increased *Akkermansia* abundance but reduced mRNA levels of inflammatory cytokines and CYP7A1. The *in vitro* study further confirmed the diet effect on gut microbiota from the middle-aged rats. Our results gave a new insight into the negative impact of fish oil diet on health of middle-aged populations by changing gut microbiota and inducing inflammation more potentially as compared to soybean oil and lard diets. Further studies will focus on the underlying molecular mechanism on how dietary fats shape gut bacteria.

## Materials and Methods

### *In vivo* study

#### Animals and diets

Thirty-three male Sprague–Dawley rats (12 months of age, 800 g ± 5 g) were bought from the Academy of Military Medical Sciences, Beijing. China. The rats were housed individually in a specific pathogen-free animal center (SYXK < Jiangsu > 2011-0037). All rats that were maintained under a 12/12 h light/dark cycle at a temperature- and humidity-controlled room (20.0 ± 0.5 °C; 60 ± 10% relative humidity) and had *ad libitum* access to one of the three fat diets and water (n = 11 for each diet group) for 3 months.

Animal diets were prepared by Jiangsu Teluofei, Inc. (Nantong, China) with soybean oil, fish oil and lard according to the formulation of the American Institute of Nutrition for middle-aged rats (AIN-93M)^55^. Soybean oil was bought from Shanghai Jiali Oil Industry Co. Ltd (Shanghai, China). Lard was obtained from Tianjin Lihongde Fat Products Inc. (Tianjin, China) and fish oil was bought from Rongcheng Ayers Ocean Bio-technology Co, Ltd (Weihai, China). The diets were composed of 4.0% oil, 14.0% protein, 46.6% cornstarch, 15.5% dextrinized cornstarch, 10.0% sucrose, 5.0% fiber, 3.5% mineral mix (AIN-93-M), 1.0% vitamin mix (AIN-93-VX), 0.2% L-cystine, 0.3% choline bitartrate, and 0.0008% tert-butylhydroquinone.

Experimental protocol involving animals was reviewed and approved by the Ethical Committee of Experimental Animal Center of Nanjing Agricultural University. All experiments were performed in accordance with the relevant guidelines and regulations of our Ethical Committee.

#### Sample collection and DNA extraction

At the end of feeding experiment, all rats were decapitalized. Liver tissue and ileal contents were also taken. Caecal and colonic contents were collected and divided into two parts. One part was immediately used for *in vitro* fermentation, and the other part was snap-frozen in liquid nitrogen and then stored at −80 °C until analysis. Microbial DNA was extracted from caecal and colonic contents by using the QIAamp DNA Stool Mini kit (NO. 51504, Qiagen, Germany) according to the manufacturer’s protocols.

### Microbiota analysis

#### PCR

To profile the microbial composition of caecal and colonic contents, all DNA samples were amplified and sequenced. The V4 region of 16S rRNA genes was analyzed. The forward primer was 515F: GTGCCAGCMGCCGCGGTAA and the reverse primer was 806R: GGACTACHVGGGTWTCTAAT. All PCR reactions were carried out in 30 μL reactions with 15 μL of Phusion® High-Fidelity PCR Master Mix, 0.2 μM of forward and reverse primers, and about 10ng template DNA. Thermal cycling consisted of initial denaturation at 98 °C for 1 min, 30 cycles of denaturation at 98 °C for 10 s, annealing at 50 °C for 30 s, elongation at 72 °C for 60 s and maintenance at 72 °C for 5 min. PCR products were adjusted to the same concentration.

PCR products were purified with GeneJET Gel Extraction Kit (Thermo Scientific). Sequencing libraries were generated using NEB Next® Ultra™ DNA Library Prep Kit for Illumina (NEB, USA) following the manufacturer’s recommendations and index codes were added. The library quality was assessed on the Qubit@ 2.0 Fluorometer (Thermo Scientific) and Agilent Bioanalyzer 2100 system. Finally, the library was sequenced on an Illumina MiSeq platform and 250 bp/300 bp paired-end reads were generated.

Paired-end reads from the original DNA fragments were merged using FLASH to merge paired-end reads when at least some of the reads overlap the reads generated from the opposite end of the same DNA fragment. Paired-end reads were assigned to each sample according to the unique barcodes. Sequencing analysis was performed by UPARSE software package using the UPARSE-OTU and UPARSE-OTUref algorithms. In-house Perl scripts were used to analyze alpha (within samples) and beta (among samples) diversities. Sequences with greater than 97% similarity were assigned to the same OTUs. We picked a representative sequence for each OTU and used the RDP classifier to annotate taxonomic information for each representative sequence. Cluster analysis was preceded by principal component analysis, which was applied to reduce the dimension of the original variables using the QIIME software package that calculated both weighted Unifrac distances to evaluate beta diversity. LEfSe analysis was performed (http://huttenhower.sph.harvard.edu/galaxy/) to discover highly-dimensional biomarkers and to characterize the differences among two or more biological conditions^56^. The different features were identified on the OTU and genus levels. The three groups were set as the class of subjects. The LEfSe analysis conditions were as follows: (1) alpha value for the factorial Kruskal-Wallis test among classes was less than 0.05; (2) alpha value for the pairwise Wilcoxon test among subclasses was less than 0.05; (3) the threshold on the logarithmic LDA score for discriminative features was less than 2.0 and (4) multi-class analysis was set as all-against-all.

#### Quantification of physiological parameters

Total RNA was isolated from the colonic and liver tissues using the commercial RNeasy mini kit (Takara, Code: 9767), according to the manufacturer’s instructions, and quantified using the Nanodrop (Thermo Scientific). Single-stranded complementary DNA (cDNA) was synthesized from 2 μL 5x PrimeScript RT Master MIX (Takara, Code: RR036A), 4 μL RNA and adding RNase free water up to 10 μL according to the manufacturer’s instructions. The condition of the reverse transcription system is: 25 °C for 3 min, 45 °C for 10 min, 85 °C for 5 min, 4 °C for chilling. The relative quantification of gene expression was performed using the QuantStudio TM 6 Flex Real-time PCR system. Primers included IL-1β (forward, 5′-TGTGTGACTCGT GGGA-3′; reverse, 5′-GTCTGTGCTCTGCTTGA-3′), IL-6 (forward, 5′-ATGAA CTCCTTCTCCACAAGC-3′; reverse, 5′-CTACATTTGCCG AAGAGCCCTCAGG CTGGACTG-3′), IL-17 (forward, 5′-CTGGGACGTACCGGG TCGGT-3′; reverse, 5′-GTCTGTCGCCTGAACAACGTCT-3′), IL-18 (forward, 5′-GGTCAGTCTTTG CTATCATTCCA-3′; reverse,5′-CAGAAAGTAAGCTTGGGGAGA-3′), β-actin as control (forward, 5′-CAGGATGGCGTGAGGGAGAGC-3′; reverse, 5′-AAGGT GTGATGGTGGGAATGG-3′) and CYP7A1 (forward, 5′-GCTATTCTCTGG GCATCTCAAG-3′; reverse, 5′-GAAAGTCAAAGGGTCTGGGT-3′). Gene expression was determined using the comparative 2^−ΔΔCt^ method.

Total bile acids and taurine-conjugated bile acids in the colon were detected by using the Bile Acid Kit (E003-2, Jiancheng, China) according to the manufacturer’s protocols. Triglyceride in the ileum was measured by using the triglyceride assay kit (A110-1, Jiancheng, China) according to the manufacturer’s protocols.

### *In vitro* study

#### Medium preparation

Three milliliters of fats and 0.03 g sodium cholate were homogenized (Ultra Turrax T25 Basic, IKA Werke, Staufen, Germany) in 7 mL of phosphate buffer solution (10 mmol/L Na_2_HPO_4_-NaH_2_PO_4_, pH7.0) for 3 × 30 s at 9,000 rpm with 1 min cooling between bursts at 4 °C. To simulate gastrointestinal tract digestion, 1 mg pancreatic lipase was added in the homogenates. The digestion was kept at 37 °C for 12 h. Afterwards, the medium was prepared by mixing 9 mL of basic solution, 0.1 mL of pancreatic-lipase-treated soybean oil, fish oil or lard, 0.1 mL of vitamin phosphate buffer solution and 0.1 mL of reducing agent^57^. To create an anaerobic condition, carbon dioxide was introduced into the medium till sealing with butyl rubber stoppers and aluminum foil. The basic solution was made by adding 0.6 g KCl, 0.6 g NaCl, 0.2 g CaCl_2_·2H_2_O, 0.5 g MgSO_4_·7H_2_O, 1.46 g KH_2_PO_4_, 4.335 g Na_2_HPO_4_, 4 g glucose, 1.0 g trypticase, 10 mL trace mineral solution, 10 mL haemin solution, 10 mL fatty acid solution, 1 mL resazurin solution and 50 mL bicarbonate solution to distilled water to a final volume of one liter.

#### Inoculums

Caecal contents were collected and immediately placed in a CO_2_-filled container. The contents were diluted in five volumes of pre-warmed PBS solution (37 °C). The diluted material was then homogenized using a hand-held mixer for 30 s under anaerobic conditions and filtered through a three layer of gauze into bottles flushed with CO_2_.

### *In vitro* fermentation

The inoculums were injected into the fermentation tube by syringes. And then, the tubes were placed in the table concentrator. After 24 h fermentation, the fermentation liquid was transferred into Eppendorf tubes and stored at −80 °C for further analyses. A control was also prepared at the same conditions but no inoculum was added.

### Microbial analysis

DNA was extracted from the fermentation liquid in chloroform and isoamylalcohol mixture^58^. The microbial composition in the fermentation liquids were analyzed using the same methods as described above. PICRUSt was applied to predict metagenome functional content from the prevalence of 16S rDNA marker gene sequences in the different samples.

#### SCFAs determination

Short-chain fatty acids were detected by GC using a modified protocol^59^, including acetic, propionic, butyric, valeric, isobutyric and isovaleric acids.

#### Statistical analysis

Differences in the relative abundance of bacteria among all groups in the *in vitro* and *in vivo* studies were evaluated by factorial analysis of variance. Means were compared by Duncan’s multiple comparison. Significance level was set at 0.05 for all statistical analyses. These analyses were run under the program of statistical analysis system (SAS, 2009).

## Electronic supplementary material

Supplement
