# Erratum: Fish oil, lard and soybean oil differentially shape gut microbiota of middle-aged rats

**DOI:** 10.1038/s41598-017-04862-8

**Published:** 2017-08-10

**Authors:** He Li, Yingying Zhu, Fan Zhao, Shangxin Song, Yingqiu Li, Xinglian Xu, Guanghong Zhou, Chunbao Li

**Affiliations:** 1 Key Laboratory of Meat Processing and Quality Control, MOE, Key Laboratory of Meat Processing, MOA, Jiang Synergetic Innovation Center of Meat Processing and Quality Control, Nanjing Agricultural University, Nanjing, 210095 P.R. China; 2 School of Food Science, Nanjing Xiaozhuang University, Nanjing, 211171 P.R. China

*Scientific Reports* **7**:826; doi:10.1038/s41598-017-00969-0; Article published online 11 April 2017

The original PDF version of this Article contained an error in the order of corresponding authors. This has now been corrected in the PDF version of this Article.

