# Supplementary material for: Fish oil, lard and soybean oil differentially shape gut microbiota of middle-aged rats
Source: Sci Rep. 2017 Apr 11;7:826. doi: 10.1038/s41598-017-00969-0 (PMC5429820; doi:10.1038/s41598-017-00969-0)
Supplement: Supplementary file 1 — Supplement [file 41598_2017_969_MOESM1_ESM.pdf]

Fish oil, lard and soybean oil differentially shape gut microbiota of middle-aged rats

He Li[a], Yingying Zhu[a], Fan Zhao[a], Shangxin Song[b], Yingqiu Li[a], Xinglian Xu[a], Guanghong Zhou[a,] *, Chunbao Li[a,] *

[a]Key Laboratory of Meat Processing and Quality Control, MOE; Key Laboratory of Animal Products Processing, MOA; Jiang Synergetic Innovation Center of Meat Processing and Quality Control; Nanjing Agricultural University; Nanjing 210095, P.R. China

[b] School of Food Science, Nanjing Xiaozhuang University; Nanjing 211171, P.R. China

*Corresponding author

Dr. Chunbao Li

College of Food Science and Technology, Nanjing Agricultural University; Nanjing 210095, P.R. China

E-mail: chunbao.li@njau.edu.cn

Tel/Fax: 86 25 84395679

Dr. Guanghong Zhou

E-mail: guanghong.zhou@hotmail.com

Running title: dietary fats shape gut bacteria

Supplementary Figure 1 Principal coordinate analyses of caecal and colonic bacteria on the OTU level

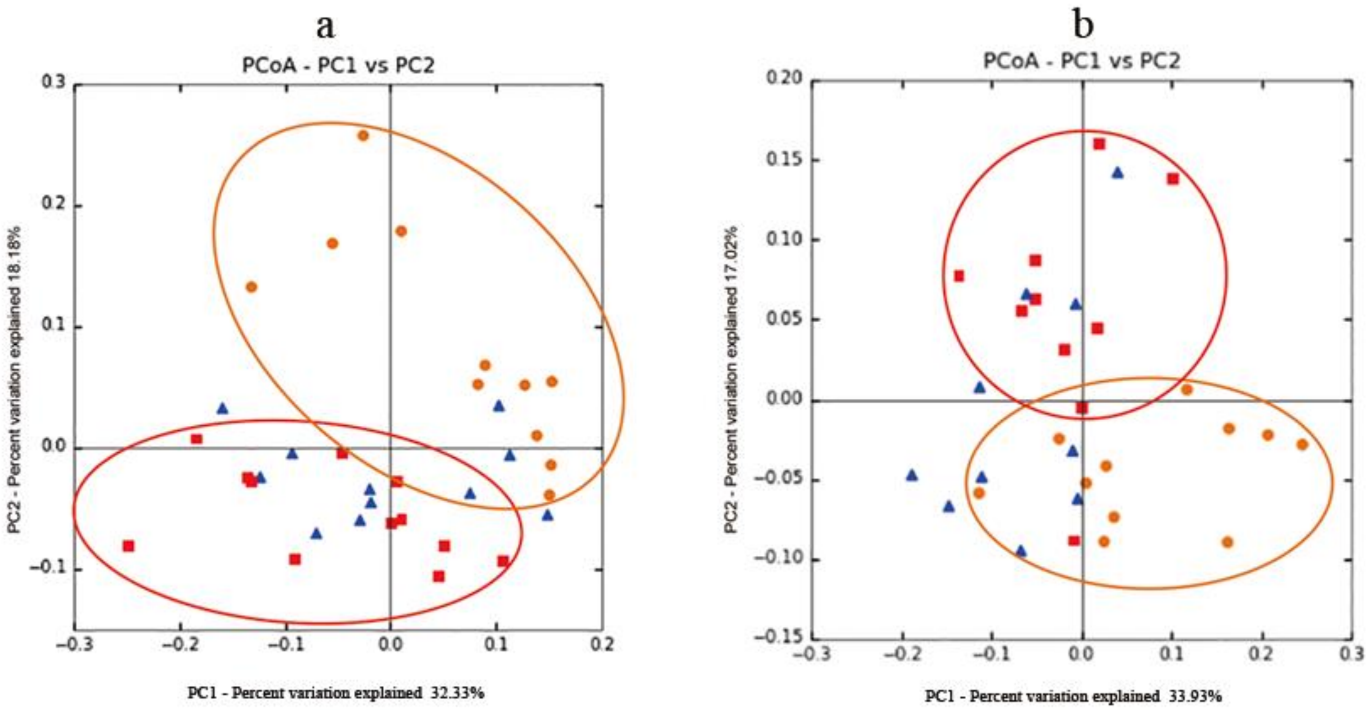

(a) caecal bacteria, (b) colonic bacteria. Orange dots, fish oil group; blue triangles, soybean oil group; red diameters, lard group.

Supplementary Figure 2 Venn plot for gut microbiota composition between in vivo caecal contents and in vitro fermentation liquids

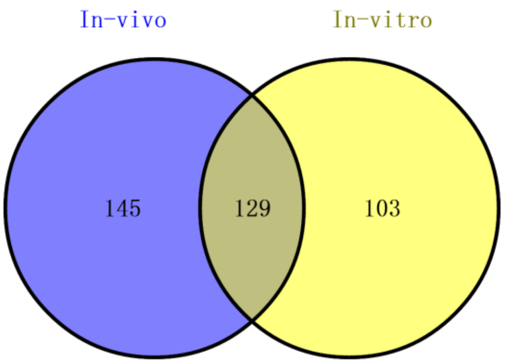

Supplementary Figure 3 PICRUSt predicted biological functions of in vitro cultivated bacteria on the third KEGG level

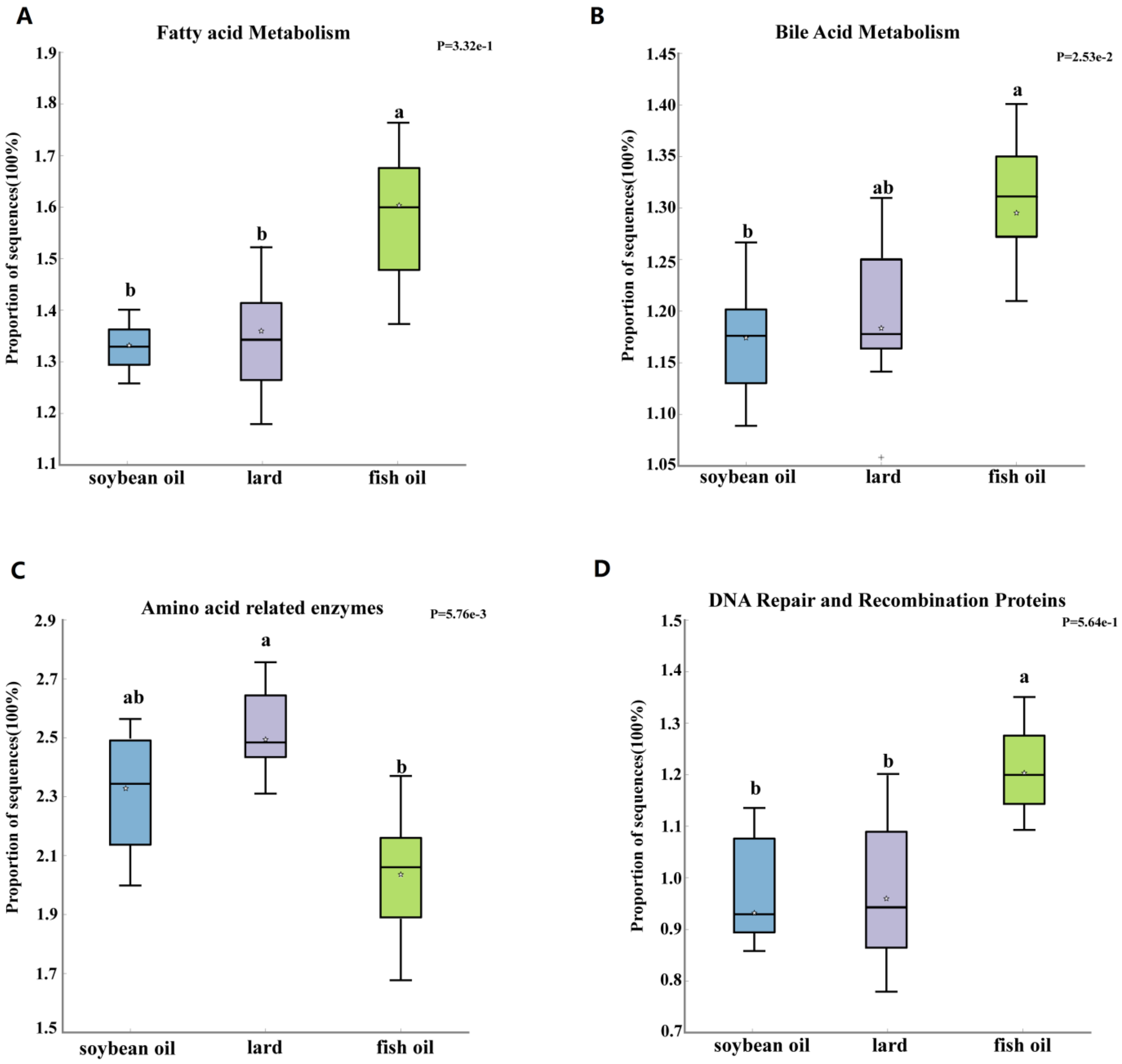

(A) Fatty acid metabolism

(B) Bile acid metabolism

(C) Amino acid related enzymes

(D) DNA Repair and Recombination Proteins

The data were analyzed by one-way analysis of variance and means were compared by the procedure of Duncan's multiple-range comparison. a,b,c, means with different letters differed significantly ($p<0.05$).

Supplementary Figure 4 PICRUSt predicted biological functions of the caecal microbiota on the second KEGG level

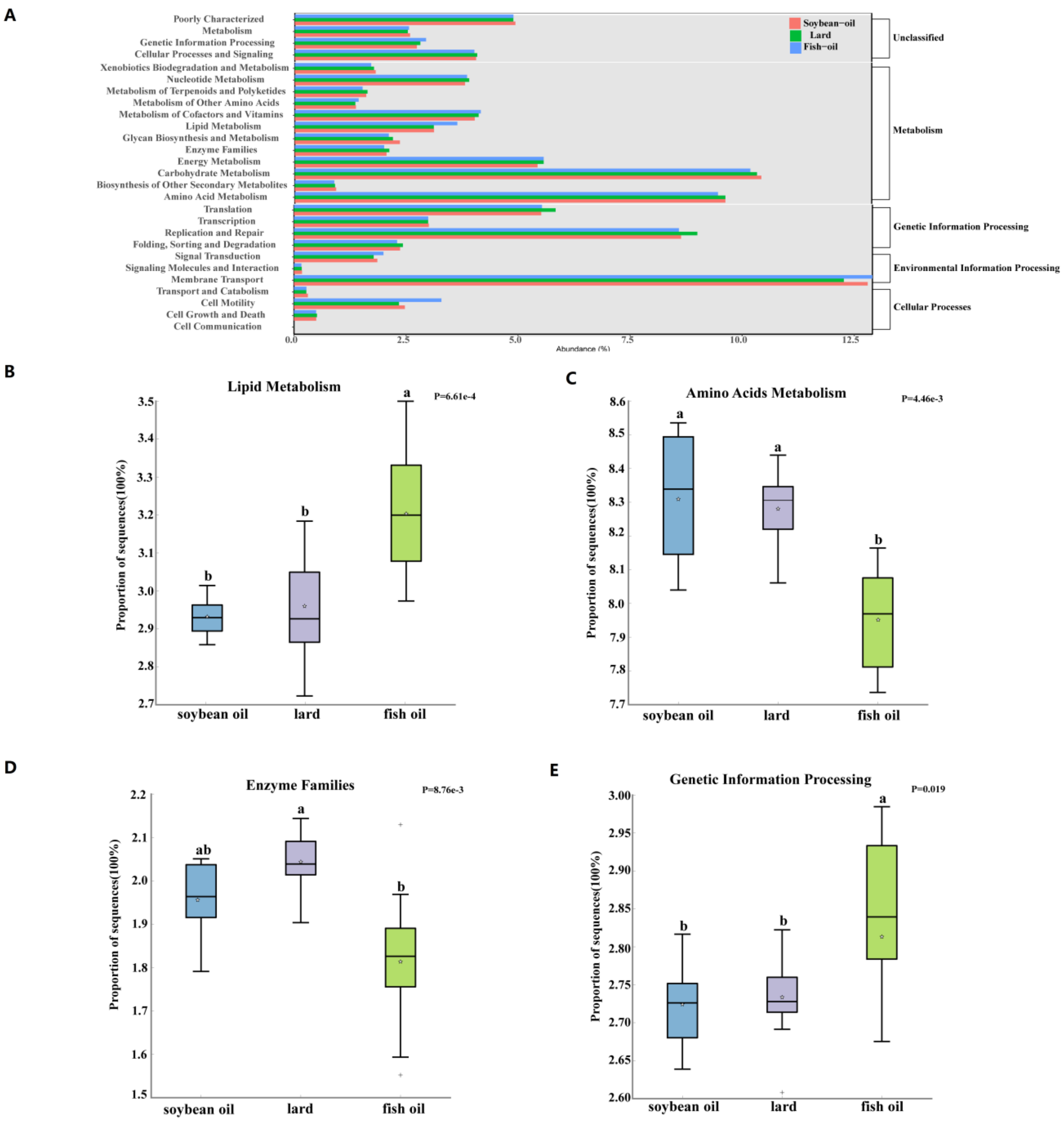

(A) Abundances of KEGG pathways in level-2 of the functional prediction by PICRUSt
(B) Lipid metabolism
(C) Amino acid metabolism
(D) Enzyme families
(E) Genetic information processing

The data were analyzed by one-way analysis of variance and means were compared by the procedure of Duncan's multiple-range comparison. a,b,c, means with different letters differed significantly (p<0.05).

Supplementary table 1 Lipid profile of oil applied in experimental diet (g/100g oil)

	Soybean oil	Fish oil	Lard
C10:0	n.d.	n.d.	0.08±0.01
C12:0	n.d.	n.d.	0.08±0.01
C14:0	n.d.	1.18±0.04	1.19±0.01
C14:1	n.d.	0.04±0	n.d.
C15:0	n.d.	0.46±0.05	0.04±0.04
C15:1	n.d.	0.15±0.01	n.d.
C16:0	10.19±0.17 [c]	20.91±0.22 [b]	24.84±0.24 [a]
C16:1	0.09±0.02 [c]	5.57±0.03 [a]	1.36±0.01 [b]
C17:0	0.06±0.01 [b]	1.07±0.22 [a]	0.29±0.01 [b]
C17:1	0.03±0.00 [b]	0.48±0.16 [a]	0.19±0.07 [b]
C18:0	4.19±0.18 [c]	6.35±0.72 [b]	15.08±0.12 [a]
C18:1n9t	n.d.	0.2±0.01	0.12±0.11
C18:1n9c	23.58±0.31 [b]	17.77±0.18 [c]	36.17±0.6 [a]
C18:2n6t	0.02±0.03	0.03±0.05	n.d.
C18:2n6c	54.06±0.4 [a]	2.4±0.03 [c]	17.19±0.44 [b]
C20:0	0.23±0.01 [b]	1.00±0.48 [a]	0.36±0.26 [b]
C18:3n6	0.6±0.02 [a]	0.3±0.02 [b]	n.d.
C20:1	0.18±0.00 [c]	3.27±0.15 [a]	0.78±0.03 [b]
C18:3n3	0.59±0.01 [c]	2.11±0.06 [a]	0.84±0.07 [b]
C20:2	n.d.	4.68±0.03 [a]	0.21±0.37 [b]
C21:0	5.87±0.07	n.d.	n.d.
C22:0	0.31±0.01 [b]	0.03±0.05 [c]	0.65±0.06 [a]
C20:3n6	n.d.	0.12±0.06	0.1±0.00
C20:4n6	n.d.	0.14±0.02 [a]	0.09±0.02 [b]
C23:0	n.d.	1.41±0.02 [a]	0.28±0.02 [b]
C24:0	n.d.	0.05±0.04	n.d.
C20:5n3	n.d.	17.35±0.39	n.d.
C24:1	n.d.	0.53±0.57	n.d.
C22:6n3	n.d.	12.41±1.54 [a]	0.07±0.00 [b]
SFA	20.86±0.29 [c]	32.47±1.20 [b]	42.87±0.10 [a]
MUFA	23.88±0.29 [c]	28±0.68 [b]	38.62±0.71 [a]
PUFA	55.26±0.41 [a]	39.53±1.75 [b]	18.50±0.65 [c]
PUFA/SFA	2.31±0.042 [a]	1.41±0.093 [b]	0.48±0.03 [c]
n6	54.67±0.40 [a]	2.99±0.04 [c]	17.38±0.46 [b]
n3	0.59±0.01 [b]	31.86±1.80 [a]	0.91±0.069 [b]
n3/n6	0.01±0.00 [b]	10.65±0.69 [a]	0.05±0.00 [b]
Cholesterol (mg/100g oil)	n.d.	121.0	96.7

Note: n.d. not detectable; [a, b, c] means with different superscripts differed significantly (P<0.05).

Supplementary table 2 Effects of different fats on feed intake and body weight gain of rats

	Soybean oil	lard	Fish oil
Feed intake（g/d）	21.74±0.96 [a]	23.31±0.81 [a]	24.12±0.64 [a]
Body weight gain（g）	96.70±6.98 [ab]	132.60±11.73 [a]	83.80±7.16 [b]
Triglyceride of the caecal contents (mmol/L)	0.31±0.13[ab]	0.44±0.12 [a]	0.21±0.09 [b]

[a,b] means with different superscripts differed significantly ($P<0.05$)
